# Selective Antitumor Activity of Datelliptium toward Medullary Thyroid Carcinoma by Downregulating RET Transcriptional Activity

**DOI:** 10.3390/cancers13133288

**Published:** 2021-06-30

**Authors:** Tariq Alqahtani, Abdullah Alswied, Daekyu Sun

**Affiliations:** 1 Department of Pharmacology and Toxicology, College of Pharmacy, University of Arizona, Tucson, AZ 85721, USA; alqahtani@pharmacy.arizona.edu; 2 Department of Pharmaceutical Sciences, College of Pharmacy, Ministry of National Guard Health Affairs, King Saud bin Abdulaziz University for Health Sciences, Riyadh 11481, Saudi Arabia; 3 Department of Pathology, University of Arizona, Tucson, AZ 85724, USA; alswied@pathology.arizona.edu; 4 The BIO5 Institute, University of Arizona, Tucson, AZ 85721, USA; 5 Department of Cancer Biology, University of Arizona, Tucson, AZ 85724, USA

**Keywords:** RET, datelliptium, NSC311152, medullary thyroid carcinoma

## Abstract

**Simple Summary:**

Medullary thyroid carcinoma (MTC) is a rare aggressive type of thyroid cancer with a propensity for metastasizing to the lymph nodes, liver, bones, and lungs. Previous studies have demonstrated that activated REarranged during Transfection (RET) mutants are key regulators of invasive and metastatic behaviors in MTC. The present study aimed to evaluate the antiinvasive and antimetastatic potential of a novel RET transcription inhibitor, datelliptium, which stabilizes the RET G-quadruplex structures and suppresses RET oncogene transcription by examining its effects on epithelial-to-mesenchymal transition (EMT), cancer stem cells (CSCs), and MTC cell migration. Interestingly, the ablation of RET with datelliptium resulted in decreased EMT, spheroid formation, and MTC cell migration. In this study, we also demonstrated the in vivo antitumor activity in TT tumor-bearing mice with about 75% tumor growth inhibition.

**Abstract:**

Medullary thyroid carcinoma (MTC) is a rare aggressive form of thyroid cancer with high rates of metastasis. Sporadic and hereditary MTC are strongly driven by somatic and germline mutations, respectively, in the transmembrane REarranged during Transfection (RET) proto-oncogene, which encodes a receptor tyrosine kinase. Our previous study identified datelliptium as a novel RET transcription inhibitor, which stabilizes the RET G-quadruplex structures and suppresses RET oncogene transcription. The present study aimed to elucidate the effect of datelliptium on the suppression of epithelial-to-mesenchymal transition (EMT) and metastasis-related behaviors of MTC cells, including cell migration and formation of cancer stem cells (CSCs). Our results demonstrated that datelliptium downregulated the expression of the mesenchymal markers, including N-cadherin, vimentin, slug, snail, and claudin-1. Compared to untreated cells, datelliptium significantly decreased the migration of TT cells in a dose-dependent manner in a wound healing assay. Additionally, datelliptium significantly reduced the size of preformed spheroids from TT cells over the time course. Finally, datelliptium inhibited approximately 75% of MTC xenograft growth with minimal systemic toxicity. In conclusion, datelliptium exerts its antitumor activity against MTC cells by reducing the EMT program, migratory ability, and self-renewal capacity of TT cells, thus preventing invasive and metastatic behavior of MTC.

## 1. Introduction

Thyroid cancer is the most common cancer of the endocrine gland neoplasm and is also the most elucidated and studied cancer [1,2]. Medullary thyroid cancer (MTC) is an aggressive form of thyroid cancer, which arises from parafollicular C-cells and comprises approximately 3–5% of all thyroid cancer malignancies [3]. Incidence of MTC involves a sporadic (non-hereditary) form in 75–80% of cases and a hereditary form in 20–25% of cases [4]. Hereditary MTC can present as an isolated neoplasm in Familial Medullary Thyroid Cancer (FMTC) or as a part of Multiple Endocrine Neoplasia Syndrome type 2 (MEN2A or MEN2B) [5]. Both sporadic and hereditary MTC are frequently associated with activating mutations in the REarranged during Transfection (RET) proto-oncogene. RET is a receptor tyrosine kinase that is encoded by the proto-oncogene RET. The activation of RET promotes cell proliferation, differentiation, and survival [6]. The oncogenic activation of RET in MTC occurs predominantly by mutations within RET that constitutively activate RET kinase, resulting in constitutive activation of downstream molecules such as MEK and ERK as well as PI3K/AKT/mTOR (PI3K) signaling pathways. This activation causes cell proliferation, invasion, and metastasis [7]. Thus, RET has become an important therapeutic target for MTC, particularly through tyrosine kinase inhibitor (TKI) therapy. Data from previous and ongoing preclinical and clinical studies investigating the efficacy of RET TKIs support the rationale that mutant RET genes are critical targets [8,9]. TKIs, such as carbozantinib and vandetanib, have been moderately successful in treatment of metastatic MTC, initially increasing cancer-specific survival times [4,10]. Recently, selpercatinib (Retevmo) has been approved by the FDA to treat MTC as well as non-small cell lung cancer and advanced RET fusion-positive thyroid cancer [11]. Another TKI, pralsetinib (BLU-667), was recently approved by the FDA for patients with advanced or metastatic RET-mutant MTC. Pralsetinib shows potent and durable clinical activity in patients with advanced RET mutation–positive MTC [12]. However, drug resistance inevitably limits the efficacy of TKIs for treatment of MTC, particularly in metastatic MTC [13]. TKIs also cause a variety of side effects in nearly all treated patients. Thus, it is critical to develop alternative therapies that may overcome resistance to and adverse effects of currently available multitargeted TKIs. 

The proximal promoter region of the RET gene consists of a guanine (G)-rich sequence containing five runs of three consecutive guanine residues, which serve as the binding site for transcription factors [14]. In the presence of monovalent cations such as Na^+^ and K^+^, G-rich sequences are able to form G-quadruplex (G4) structures, which are four-stranded structures consisting of four in-plane guanine bases held together by eight hydrogen bonds. G-rich sequences of telomeric DNA were first reported to form G4 structures, which were later found to be over-represented in promoter sequences of many oncogenes and proto-oncogenes as well as genes involved in proliferation, including c-myc, VEGF, RET, c-Kit, Kras, EGFR, PDGFR2, BRAF, and c-Src [14,15]. Interestingly, the formation of Intramolecular G4 structures within oncogene promoter regions was demonstrated to effectively inhibit oncogene transcription and expression [16].

**Figure 1:**
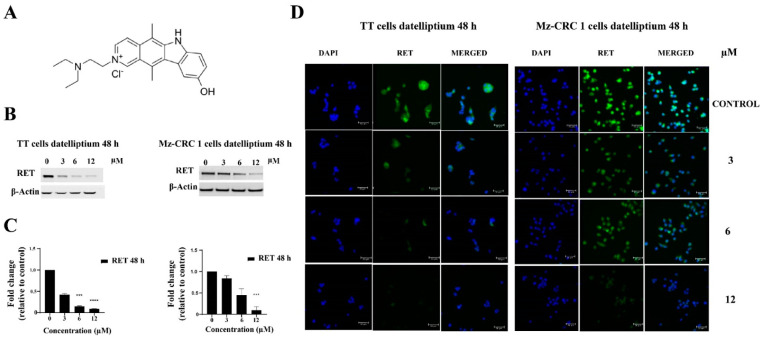
The antitumor activity of datelliptium on RET expression. (**A**) Structure of datelliptium, (**B**) Western blot analysis of the expression of RET in TT and MZ-CRC-1 cells treated with datelliptium for 48 h. (**C**) Densitometry analysis of the western blotting results for RET was normalized to its basal expression (*n* = 3, **** *p* < 0.0001, and *** *p* < 0.001) using one-way ANOVA with Tukey post-test. (**D**) Immunofluorescence staining of the expression of RET in TT and MZ-CRC-1 cells treated with datelliptium for 48 h (×400, scale  =  50 μm); DAPI (blue stained nuclei).

Our recent studies provided compelling evidence that a specific G4 structure formed in the RET promoter functions as a transcriptional repressor element and that RET transcription in MTC cells could be repressed by ligand mediated G4 stabilization [14,17]. Our previous study developed a luciferase cell-based assay with the HEK293 RETWT cell line by introducing the luciferase gene to HEK293 cells under the control of a RET promoter to discover new G4-interactive agents [14]. Our initial screening of the NCI Diversity set, and other NCI chemical libraries identified datelliptium (SR 95156B; NSC311152, Figure 1A) as a possible G4-interactive molecule as well as other G4-interactive molecules described in our previous studies [18]. Further insight into the cellular mechanisms by which G4-interactive agents inhibit RET expression has been provided by a chromatin immunoprecipitation (ChIP) assay [17]. In these studies, a ChIP assay revealed that G4-interactive compounds prevent the binding of Pol II, Sp1, and nucleolin to the RET promoter, suggesting that G-quadruplex-interactive small molecules would interfere with transcription complex assembly at the human RET gene promoter region. Remarkably, datelliptium has been evaluated in phase I trials for safety and other pharmacokinetic parameters even without knowledge of its G-4 binding property [19]. Furthermore, various G4-interactive agents have been investigated as potential antitumor agents in preclinical studies but have not yet been studied in human trials to evaluate safety [16].

Our previous study validated the RET promoter G-quadruplex as a potential target for new anti-cancer therapies against advanced and metastatic MTC [18]. In that study, we demonstrated that the anti-cancer mechanism of action of datelliptium involves stabilization of a G-quadruplex structure on the RET promoter region, leading to RET downregulation [18]. Notably, datelliptium did not suppress RET/PTC1 expression in a PTC1-derived TPC1 cell line used as a control, in which RET transcriptional activation is controlled by a CCD6 promoter region lacking a G-quadruplex forming motif due to chromosomal rearrangement between the RET kinase domain coding region and the CCD6 gene [18]. Overall, these data provide clear evidence that datelliptium interferes with RET transcription by targeting the intracellular G-quadruplex structure formed within its promoter region. In the same study, we also investigated if datelliptium treatment could affect proliferation of normal diploid cells by including a normal thyroid cell line, Nthy-ori-3-1, as a control [18]. Based on the MTS data, the IC_50_ of datelliptium in the Nthy-ori-3-1 cell line was estimated to be 10 µg/mL, four-fold higher than that for TT cells, suggesting that this compound is selectively toxic against mutant RET-driven thyroid cancer. We also reported promising in vivo antitumor activity by datelliptium using MTC xenograft mouse models [18]. Although it has been demonstrated that datelliptium exhibits various biological processes in MTC cells, the potential anti-tumor mechanisms remain to be fully elucidated. The major causes of death in patients with MTC include metastasis, acquired resistance to targeted therapy, and limited response to conventional chemotherapy and radiation therapy [3]. Epithelial-mesenchymal transition (EMT) is known to play a critical role in the development and invasiveness of MTC; therefore, EMT pathways could be main targets for novel drug development for MTC [20,21,22]. In light of this, it is of great clinical importance to investigate the potential effects of datelliptium in patients with MTC. Oncogenic RET is closely related to EMT in MTC as both PTC isoforms and MTC-associated RET mutants can induce the expression of EMT-related genes and EMT-associated biological activities [21,22]. In this study, we aimed to investigate the effects of datelliptium on EMT, spheroid formation, and cell migration in MTC cell lines. Our results demonstrate that datelliptium can reverse EMT and inhibit RET expression in MTC cells.

## 2. Materials and Methods

### 2.1. Chemicals

Datelliptium (NSC311152) was kindly provided by the U.S. NCI/DTP Open Chemical Repository. Datelliptium was dissolved in dimethyl sulfoxide (DMSO) at a final concentration of 10 mg/mL.

### 2.2. Cell Culture and Media

The human medullary thyroid carcinoma (TT) and MZ-CRC-1 cell lines were obtained from the American Type Culture Collection (Manassas, VA, USA). The MTC cell line TT carries a Cys634 → Trp in exon 11 of the RET gene, while Mz-CRC1 carries a Met918 → Thr in exon 16 of the RET gene. Both mutations activate RET without a soluble ligand of the glial cell-line-derived neurotrophic factor (GDNF) family and a co-receptor of the GDNF family receptors α (GFRα) [6,7]. Cell authentication was completed for both cell lines. TT and MZ-CRC-1 cells were cultured in Dulbecco’s Modified Eagle’s Medium (DMEM/F12) and DMEM (Cellgro; Manassas, VA, USA), respectively, with 15% heat inactivated fetal bovine serum (FBS). All cells were maintained in a humidified atmosphere containing 5% CO_2_ at 37 °C. All cells were tested negative for mycoplasma contamination.

### 2.3. Western Blotting

After 24 or 48 h of treatment with datelliptium, whole-cell extracts were prepared from harvested cells or tumor tissues, and protein concentration in cell lysates was measured by using Bio-Rad Protein Assay Dye Reagent (Bio-Rad Laboratories, Inc., Hercules, CA, USA) as previously described [23]. Total proteins were separated by either 4–12% or 12% gradient polyacrylamide SDS-PAGE, as described previously [23]. The primary antibodies used were: Akt (#9272), pAkt (Ser473, #9271), mTOR (#2983), phosphorylated (p)-mTOR (#5536), RET (#3220), retinoblastoma (Rb; #9309), p-Rb (#8516S), ERK1/2 (#8544), and pERK1/2 (#3192) (dilution 1:1000) purchased from Cell Signaling Technology, Inc.; cyclin D (sc-20044) was purchased from Santa Cruz Biotechnology, Inc (Danvers, MA, USA). Secondary antibodies, either mouse or rabbit IgG antibodies conjugated with HRP (#1706516 and #1706515 Bio-Rad Laboratories, Inc.), were used (dilution 1:1000). For signal detection, an enhanced chemiluminescence substrate kit (#32106; Thermo Fisher Scientific, Inc. Waltham, MA, USA) was used. Densitometric analysis was performed using Image J software (version 1.51) and protein expression was normalized to β-actin. The original western blots can be found in Figure S1.

### 2.4. Immunofluorescence Analysis

TT or Mz-CRC-1 cells were seeded on the coverslip overnight. After treatments, cells were fixed by 4% paraformaldehyde in PBS for 15 min and then permeabilized with Triton X-100 in PBS for 10 min at room temperature. BSA (2%) in PBS was used as a blocking buffer. After 1 h, cells were incubated with primary antibodies for 2 h at room temperature or overnight at 4 °C. Samples were washed with 1X PBS then incubated for 2 h with secondary antibodies at room temperature in the dark. Slides were stained with DAPI and washed with PBS several times. Slides were mounted with coverslips for fluorescence microscopy analysis. Images were captured with inverted Leica DMI6000 (Leica, Allendale, NJ, USA).

### 2.5. Wound Healing Scratch Assay

The migration ability of TT cells was tested by using wound healing scratch assay. TT cells were seeded in 12 well plates. After 24 h, cells reached 70–80% confluency and were scratched by a sterile 200 μL pipette tip. Cells were washed with 1X PBS to remove cellular debris. Cells were incubated with either datelliptium or vehicle up to 96 h. Scratched areas were photographed under an inverted microscope. Relative covered area % were measured by Image J software (version 1.51).

### 2.6. Spheroid Formation

TT spheroids were generated by seeding about 7000 cells/well in ultra-low attachment (ULA) 96-well round-bottomed plates (Corning, NY, USA) as per manufacturer’s protocol. Cells were treated with either vehicle or datelliptium and photographed under an inverted microscope.

### 2.7. Tumor Xenograft Study

Male SCID (severe combined immunodeficiency) mice (8–10 weeks old) were used to study the efficacy of datelliptium treatment in vivo. TT cells were injected subcutaneously into the flank of the mice and the tumor growth was closely monitored every 3 days by measuring and recording tumor diameter by using a vernier caliper. Tumors were measured 3 times a week during the treatment. The volume of tumors was assessed by using the formula: tumor volume = (length × width^2^)/2, where length represents the largest tumor diameter and width represents the perpendicular tumor diameter. Once the tumor volume reached 100 mm^3^, the mice were randomly pair matched to a treated group and control group (*n* = 6/group). Datelliptium was dissolved in 90% PBS and 10% DMSO and administered intraperitoneally as a single dose of 6 mg/kg for 5 days/week for 3 weeks. Relative tumor volume was calculated by using the formula: relative tumor volume = Tx (absolute tumor volume on day X) × 100/(absolute tumor volume of same tumor on day 0). The toxicity of the compound was evaluated based on the loss of average weight of mice. At the end of the treatment, tumor tissue from control and treated mice were obtained for further analysis by western blotting. All the animal experiments were conducted in accordance with the Institutional Animal Care and Use Committee (IACUC) 07-029 approved on 22 February 2016. The experiments were completed in the Experimental mouse shared resource (EMSR) animal facility laboratory (University of Arizona, Tucson, AZ, USA), which is accredited by the international Association for Assessment and Accreditation of Laboratory Animal Care (AAALAC).

### 2.8. Statistical Analysis

All data were reported as the standard error of the mean (±SEM) of at least three independent experiments. For the determination of significant differences among multiple treatment groups, we utilized one-way ANOVA with Tukey’s post hoc test or two-way ANOVA with Bonferroni post hoc analysis. Data were statistically analyzed using a Student’s t-test; when *p* < 0.05, the difference between groups was considered to be statistically significant. GraphPad Prism V.8 (GraphPad Software, Inc., San Diego, CA, USA) software was utilized for all data analysis.

## 3. Results

### 3.1. Effect of Datelliptium on RET Protein Expression in MTC Cell Lines

Our previous studies have revealed that datelliptium selectively downregulates RET expression levels in MTC cells [18]. In this study, western blot and immunofluorescence analyses were used to detect RET protein expression in two representative MTC cell lines treated with datelliptium for 48 h. As shown in Figure 1B,C, western blot analysis revealed that RET is downregulated in TT and Mz-CRC 1 cell lines. Immunofluorescence staining with a primary antibody against RET also confirmed downregulation of RET expression in two MTC cell lines harboring RET mutations when treated with datelliptium for 48 h (Figure 1D).

### 3.2. Effects of Datelliptium on EMT in TT Cells

**Figure 2:**
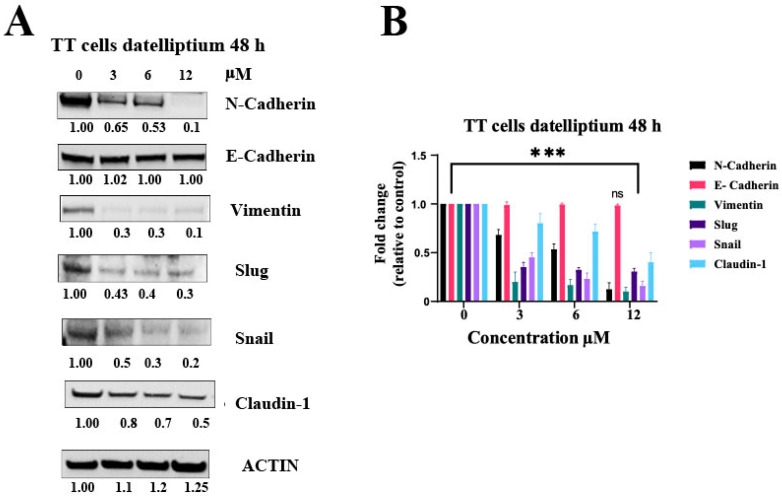
Inhibition of the epithelial-mesenchymal transition in TT cells by datelliptium. (**A**) The levels of each protein were analyzed by western blot after treatment with 3, 6, and 12 μM of datelliptium. (**B**) Densitometry analysis of the western blotting in A; all proteins were normalized to their basal expression (*n* = 3, *** *p* < 0.001, and ns = not significant) using one-way ANOVA with Tukey post-test.

EMT has been implicated in metastasis for transforming relatively non-invasive epithelial cancer cells into highly invasive mesenchymal cells [24]. Through this process, tumor cells are known to acquire mesenchymal features to become invasive, losing their epithelial characteristics. These morphological and molecular changes are accompanied by increased expression of a set of pleiotropically acting transcription factors (Snail1, Slug, Twist1, and ZEB1). These transcription factors are frequently expressed during EMT leading to invasion, dissemination, and metastasis [25]. Vimentin is a type III intermediate filament (IF) protein that is expressed in mesenchymal cells [26]. Claudin-1 is known to promote EMT by activating the Wnt/β-catenin signaling pathway [27,28]. Gain of N-cadherin and loss of E-cadherin is considered a hallmark of thyroid cancer metastasis [29]. These EMT-related genetic changes are inducible by activating RET MEN2 mutants both in cell cultures and in MTC samples as shown by differential display and microarray analysis [22,30]. In a previous study, silencing RET gene expression using siRNA suppressed EMT in papillary thyroid carcinoma cells has also been reported [31]. Since the activation of EMT will allow carcinoma cells to dissociate from each other for single-cell migration and invasion, EMT is believed to be a major way for cancer cells to acquire migratory capacity. As shown in Figure 2A,C, datelliptium downregulates the expression of the mesenchymal biomarkers, including N-cadherin, vimentin, Slug, Snail, and cladin-1 in a dose-dependent manner. This result demonstrates the anti-EMT effect of datelliptium against TT cells.

### 3.3. Effects of Datelliptium on Migration in MTC

**Figure 3:**
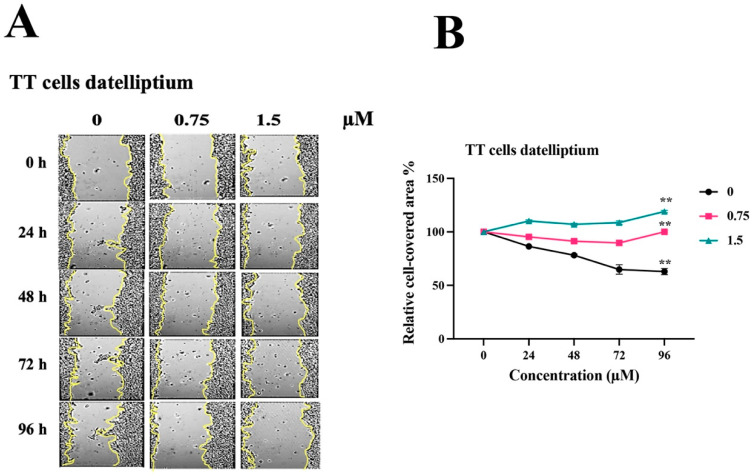
Effects of datelliptium on the migration of TT cells. (**A**) A narrow scratch was created in a confluent monolayer of TT cells growing on the bottom of a 12-well plate and incubated at 37 °C for 96 h. (**B**) Relative cell covered area analysis of wound closure in (**A**). (*n* = 3, ** *p* ≤ 0.01).

In order to invade and metastasize to internal organs, active migration of tumor cells is essential [24]. The RET receptor plays a pivotal role in regulating cell migration, proliferation, and invasion of MTC [21,32,33]. Thus, we determined the migratory ability of TT cells treated with datelliptium at various doses using the scratch (wound healing) assay according to the published procedure [34]. When TT cells were subjected to a scratch assay, it took approximately 96 h for the gap to close to 50% of its original area as shown in Figure 3A. When treated with datelliptium, TT cell migration was completely stopped at more than 0.3 µg/mL concentrations (Figure 3A). These results suggest that datelliptium is very effective in blocking TT cell migration in a scratch assay.

### 3.4. Effects of Datelliptium on Preformed CSCs from TT Cells

**Figure 4:**
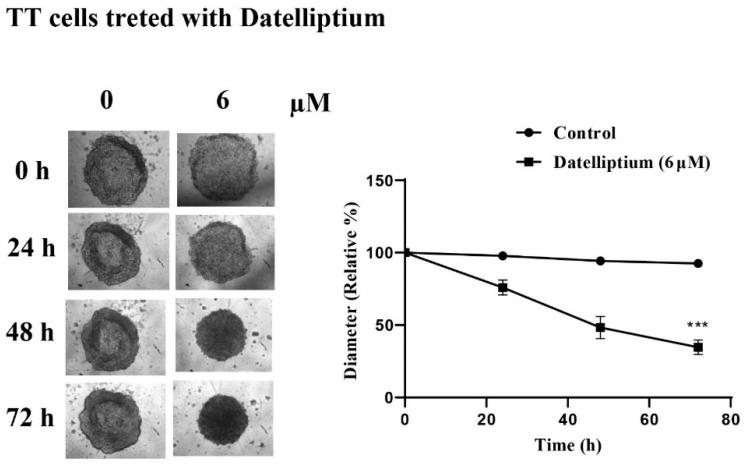
Effect of datelliptium on the growth of spheroids from TT cells. Spheroids were formed by plating about 7000 cells and were allowed to grow for 2 days prior to treatment with datelliptium. Day 0 shows the start of treatment following the initial 2-day growth period. Images are shown for spheroids treated with 0 and 6 µM of datelliptium. Spheroid growth and reduction were tracked over 4 days. The relative diameter of the spheroid was measured using ImageJ (*n* = 3, *** *p* < 0.001).

CSCs exhibit an important function in MTC malignant progression, therapeutic resistance, and recurrence [35]. The RET signaling pathway is known to be involved in stem cell maintenance as well as the development of MTC [30,36,37]. Since the ability to form tumor spheroids is an in vitro indication of the presence of CSCs, we initially monitored the formation of TT spheroids in non-adherent serum-free culture medium. As shown in Figure 4, TT cells were able to form spheroids. Next, we examined the effects of datelliptium on the size of preformed spheroids from TT cells. As shown in Figure 4, we observed a significant decline in spheroid size over the time course. Cell death proceeded from the periphery of the spheroid inwards. While spheroid volume of untreated cells was stabilized, cell density within the spheroid was increased. Even after 48 h incubation, the diameter and volume of the spheroids were reduced in the presence of datelliptium from pre-treatment starting values by up to 32%, 52%, and 70%, respectively.

### 3.5. Effects of Datelliptium on Cyclin D1 Expression in TT and MZ-CRC-1 Cells

**Figure 5:**
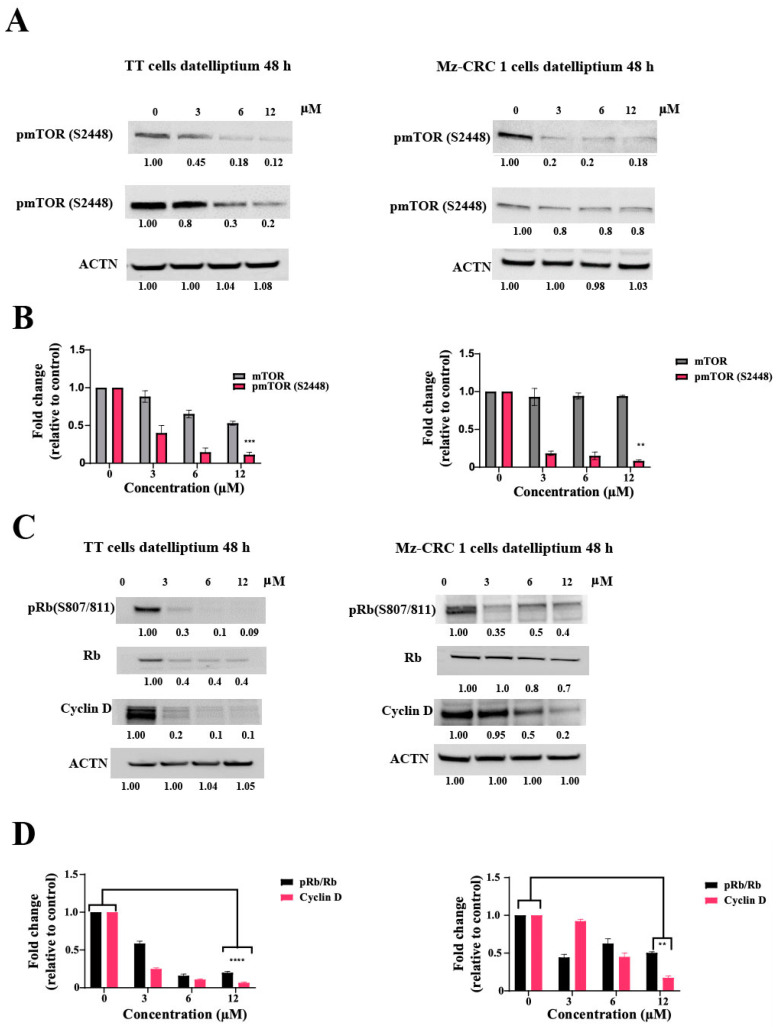
Datelliptium downregulates cyclin D1 protein expression levels in TT- and MZ-CRC-1 cells. (**A**) Cells were treated with datelliptium for 48 h at several concentrations, and the levels of phosphorylated and total mTOR protein were measured by western blotting. (**C**) TT- and MZ-CRC-1 cells were treated for 48 h with indicated concentrations of datelliptium and harvested to get protein lysates. The cellular lysates prepared from TT- and MZ-CRC-1 cells were analyzed by western blot using specific antibodies against cyclin D1, Rb, or pRb protein. Actin served as a control for equal loading. (**B**,**D**) Densitometry analysis of the western blotting of (**A**,**C**), respectively. pmTOR, mTOR, cyclin D, and pRb/Rb were normalized to their basal expression (*n* = 3, **** *p* < 0.0001, *** *p* < 0.001, and ** *p* ≤ 0.01).

Cyclin D1 is one of the most important human oncogenes and believed to be involved in the pathogenesis and metastatic potential of thyroid tumors [38,39]. Our current study revealed that datelliptium reduced the activation of the PI3K/Akt/mTOR pathway in TT and Mz-CRC1 cells by downregulating RET expression (Figure 5A,B). Since mTOR exerts its regulatory effects on cell proliferation by increasing cyclin D1 levels, the effects of datelliptium on cyclin D1 expression was examined in both TT- and MZ-CRC-1 cells [39,40]. As shown in Figure 5C,D, the cyclin D1 protein was significantly downregulated in both cell lines treated with datelliptium. Cyclin D1 in complex with CDK4 and CDK6 phosphorylates the retinoblastoma (Rb) protein (Rb) to facilitate the progression from G1 to S phase. A decrease in cyclin D1 reduces the levels of mono-phosphorylating Rb in both cell lines treated with datelliptium (Figure 5C,D). This result suggests that datelliptium effectively blocks the inactivation of the retinoblastoma protein (pRb) by phosphorylation.

### 3.6. In Vivo Antitumor Activity of Datelliptium in a MTC Xenograft Model

**Figure 6:**
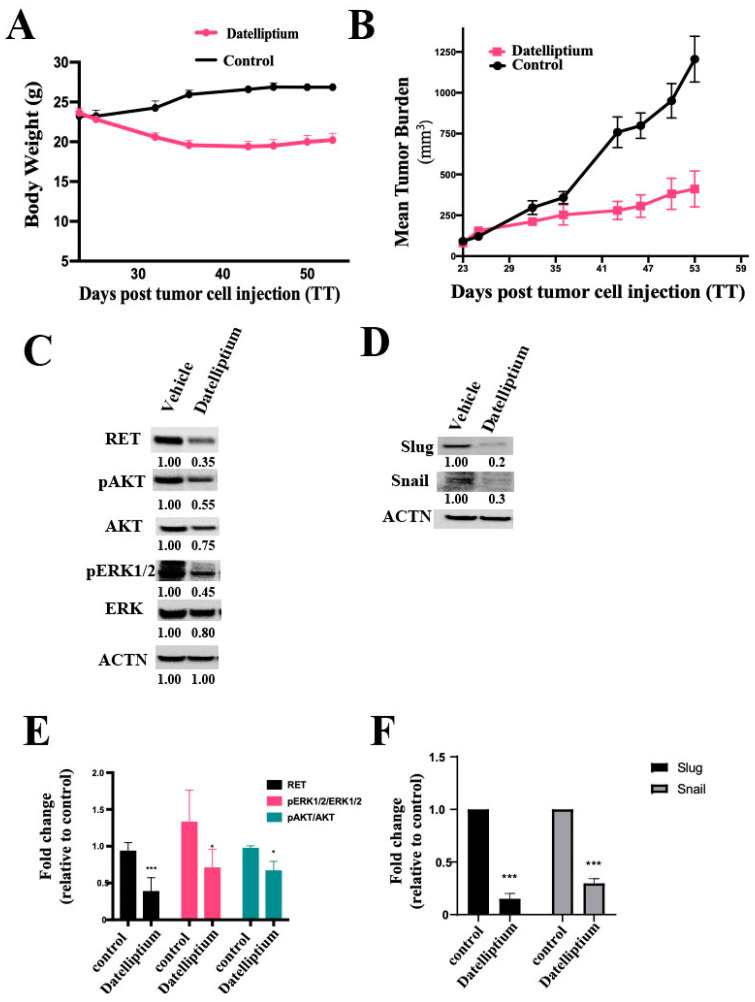
Effect of datelliptium on medullary thyroid tumor growth in vivo. (**A**) Average weight of mice in the control group and datelliptium (6 mg/kg) treated group; x- and y-axes illustrate days post-treatment and the average weight of mice, respectively. (**B**) MTC tumor growth curves of the vehicle treated group and datelliptium (6 mg/kg) treated group; x-axes and y-axes represent the number of days after drug administration and the average tumor volume, respectively. Data are mean ± SEM of 6 different mice. (**C**) Western blot analysis to evaluate RET, AKT, pAKT, pERK1/2, ERK1/2, and actin expression of the tumor tissues post-treatment. (**D**) Western blot analysis to selected EMT biomarker Slug and Snail. (**E**,**F**) Densitometry analysis of (**C**,**D**), respectively. All proteins were normalized to their basal expression (*n* = 3, *** *p* < 0.001, and * *p* ≤ 0.5).

In our previous study, this compound exhibited a moderate antitumor effect in vivo, resulting in approximately 50% tumor growth inhibition with i.p. injections of datelliptium at 4 mg/kg for three weeks (5 consecutive days/week) [18]. Thus, a dose-escalation experiment with datelliptium was needed to optimize dosage for balancing therapeutic response and side effects in the treatment of MTC xenografts derived from TT cell lines. In the present study, the dose of datelliptium was escalated approximately 1.5-fold (6 mg/kg) compared to the doses used in our previous study. Treatment of TT tumor-bearing mice with i.p. injections of vehicle or datelliptium at 6 mg/kg for four weeks (5 consecutive days/week) resulted in approximately 70% tumor growth inhibition (Figure 6B). Furthermore, treatment with datelliptium did not show toxicity or weight loss at four weeks post-treatment (Figure 6A). Consistent with findings from in vitro studies, datelliptium reduced pAKT, pERK1/2, and RET expression in vivo (Figure 6C,E). Overall, these data suggest that datelliptium achieves potent in vivo anticancer activity through RET inhibition. Additionally, western blot analysis of protein levels of slug and snail in tumor specimens treated with datelliptium revealed that it could inhibit EMT in vivo (Figure 6D,F).

## 4. Discussion

Repurposing an existing investigational drug as a potent anticancer agent for MTC therapy presents important advantages compared to investigating newer agents. One such advantage is the opportunity to bypass additional phase I safety and dosing clinical trials [39]. TKI-associated limitations, including drug resistance and adverse effects, are frequently reported in patients with MTC, and no current drug serves as a cure for advanced MTC. Initial tumor stabilization or regression can be induced by TKIs including cabozantinib and vandetanib, but positive effects are not sustained. Resistance to TKIs is considered inevitable, even with more potent second- and third-line therapies or new agents in development [4,40]. TKIs also cause varied side effects in almost all treated patients, including hypertension, gastrointestinal disturbances, thyroid reactions, fatigue, and weight loss [41]. If adverse events appear, dose reduction, temporary treatment interruption (drug holiday), or treatment discontinuation is necessary. However, each of these approaches leads to disease relapse and progression [42]. The clinical limitations of TKI monotherapy justify the investigation of additional therapeutic strategies, especially in patients with advanced MTC. To date, extensive effort has been devoted to developing novel TKIs; however, very little effort has been devoted to developing new therapeutic strategies that can overcome the limitations of small molecule TKIs. A paradigm shift in MTC treatment is needed to prioritize the development of new and more effective first-line treatment approaches including strategies aimed at suppressing oncogenic functions by means other than catalytic inhibition of RET.

Metastatic disease is a major clinical challenge in the treatment of MTC, one of the most aggressive and deadly forms of thyroid cancer, due to its propensity to metastasize to the lymph nodes, liver, bones, and lungs [43]. The stemness of cancer cells, EMT, migration, invasion, and production of factors that shape the tumor microenvironment are considered major indicators of invasive and metastatic behaviors in human cancer, including MTC, under in vitro and in vivo conditions [30,44,45]. Our results described in the present study suggest these processes can be reversed in MTC cells by treatment with datelliptium. More specifically, we demonstrated that spheroid formation, migration, and invasion can be reversed in MTC cells by treatment with datelliptium. Mutations in the RET proto-oncogene are also known to activate the phosphatidylinositol 3-kinase (PI3K)/Akt/mammalian target of rapamycin (mTOR) pathway, which is a central hub for the regulation of cell proliferation, apoptosis, cell cycle, metabolism, and angiogenesis [23]. mTOR exerts its regulatory effects on cell proliferation primarily by increasing the production of cyclin D1 [38]. Cyclin D1 overexpression has been associated with a number of cancers including MTC [46,47]. Several recent studies suggest that cyclin D1 is involved in sustaining the mesenchymal feature of CSC-like cells in EMT [48,49]. The silencing cyclin D1 was found to decrease cell proliferation and migratory capability by downregulating the expression of the mesenchymal markers such as vimentin and N-cadherin [48,49]. It is important to note that cyclin D1 was significantly downregulated in both MTC cell lines treated with datelliptium.

Datelliptium was studied in phase I trials in escalating doses given on a single 1h or 24 h continuous intravenous infusion schedule for patients with different cancers, including breast, squamous lung, ovarian, and other forms [19]. Phase I clinical trials did not include thyroid cancer patients. While patients achieved only a minor response and long-term disease stabilization at best, neither drug-related deaths nor hematological toxicity and mucositis were observed at doses of >200 mg/m^2^. No noticeable toxic effects were encountered up to 84 mg/m^2^/day. Clinical adverse events included moderate nausea and vomiting, mild diarrhea, dry mouth, neuropsychiatric manifestations, and fatigue at doses ≥330 mg/m^2^. Side effects were reversible and did not require dose limiting. The human MTD of datelliptium is estimated to be around 500 mg/m^2^/day or 14 mg/kg/day, assuming an average body weight of human adult patients in two phase I trials of 60 kg. Other studies suggest that mice can tolerate up to 30–35 mg/kg i.p. daily [19,50,51,52]. As shown in Figure 6, our study demonstrated that datelliptium was active against MTC in mouse models even at less than 6 mg/kg i.p. once a day (days 1–5 per week for four weeks), which is equivalent to 20 mg/m^2^ for humans based on body surface area. Although complete regression was not observed in our study, approximately 75% of tumor growth inhibition was achieved when datelliptium was administered as monotherapy at a dose of 6 mg/kg. Thus, we anticipate that short-term treatment (four weeks) with datelliptium, particularly at this low dose, will not produce noticeable human toxicity.

Datelliptium can be used in monotherapy to prevent disease relapse or progression for a specific population of patients facing treatment interruption or discontinuation caused by drug resistance or severe adverse effects. Datelliptium can be used in combination with TKIs to prevent disease relapse or progression for patients who need dosage reduction due to side effect intolerance of the TKIs. Datelliptium demonstrates a high potential to be repurposed with fast-track approval as a new therapeutic option for MTC patients experiencing TKI resistance and/or severe adverse side effects who lack other available therapeutic options. New TKIs (selpercatinib and pralsetinib) are better tolerated than predecessor drugs such as cabozantinib and vandetanib. However, eventual dose reduction is likely for most patients because of treatment-related adverse side effects and/or resistance [53]. Combining existing TKIs with datelliptium, which offers a different mechanism of action, may enhance antitumor effects beyond the use of TKIs alone. In future studies, the combination of selpercatinib or pralsetinib with datelliptium should be tested to determine if such combinations offer superior antitumor effects compared to TKI monotherapy in preclinical in vitro and in vivo models of MTC.

As with any treatment approach, there are potential drawbacks to the use of datelliptium. Although there are no currently reported point mutations known to affect G4 stability in the RET promoter region, the potential development of such mutations could result in the emergence of drug resistance to datelliptium. We plan to address this issue in future studies by establishing datelliptium-resistant MTC cell sublines and determining if acquired datelliptium resistance is associated with mutations of the G4-forming region in the RET promoter. Our ongoing study is focused on using transcriptome sequencing to determine whether datelliptium exclusively affects RET transcription, since G4 motifs have also been identified at the promoter regions of many other proto-oncogenes. Our preliminary analysis has confirmed RET as a major target for datelliptium. However, BRAF has been identified as another potential target for datelliptium, so we are currently investigating this issue (data not shown).

## 5. Conclusions

EMT status has been correlated to the metastatic behavior and survival outcomes of MTC. Based on our results described in the present study, these processes can be reversed in MTC cells by treatment with datelliptium. More specifically, we demonstrated that spheroid formation, migration, and invasion can be reversed in MTC cells by treatment with datelliptium. In summary, the results of our study provide new information on the therapeutic potential of datelliptium for the treatment of MTC, replacing or complementing current therapies employing TKIs to extend the lifespan of MTC patients and improve their quality of life.

## Data Availability

All data generated or analyzed during this study are included in this published article.

## Supplementary Materials

The following are available online at https://www.mdpi.com/article/10.3390/cancers13133288/s1, Figure S1: original western blots.
