# Supplementary material for: Selective Antitumor Activity of Datelliptium toward Medullary Thyroid Carcinoma by Downregulating RET Transcriptional Activity
Source: Cancers (Basel). 2021 Jun 30;13(13):3288. doi: 10.3390/cancers13133288 (PMC8267783; doi:10.3390/cancers13133288)
Supplement: Supplementary file 1 [file cancers-13-03288-s001.zip › Figure S1.pdf]

Figure 1

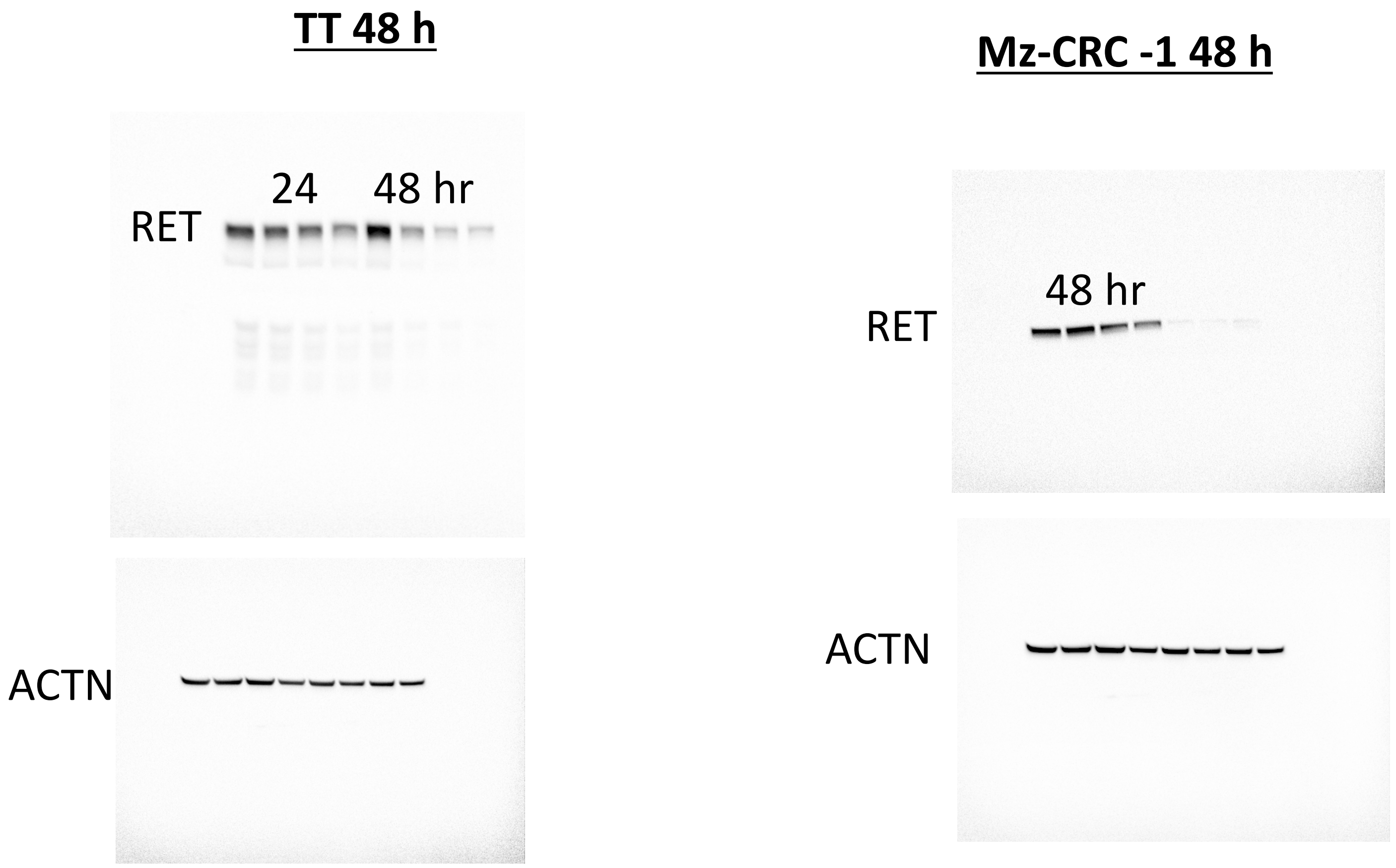

Figure 2

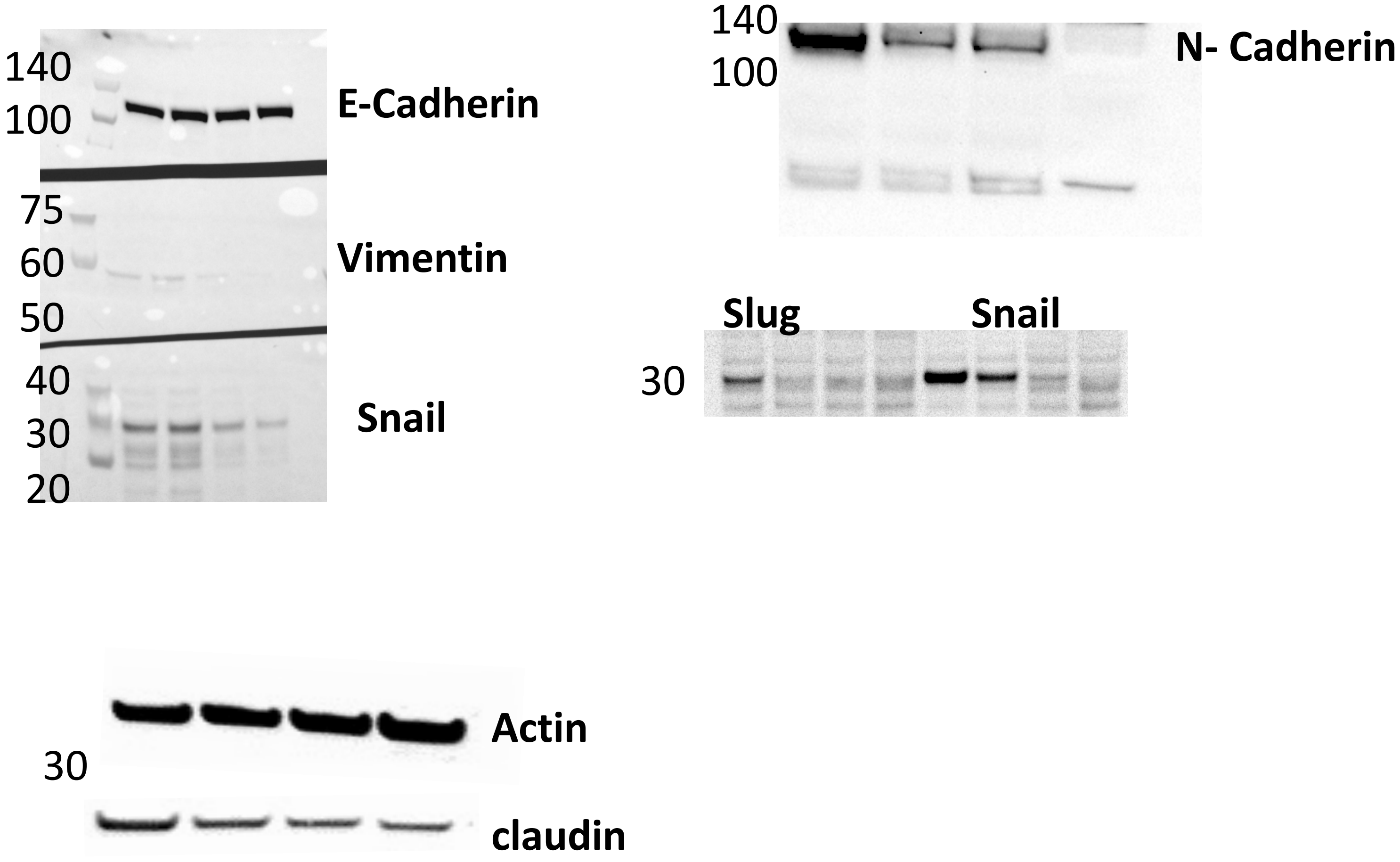
140
100
E-Cadherin
75
60
50
Vimentin
40
30
20
Snail
140
100
N- Cadherin
Slug
Snail
30
Actin
30
claudin

Figure 5A for TT 48 hours

TT 48 h

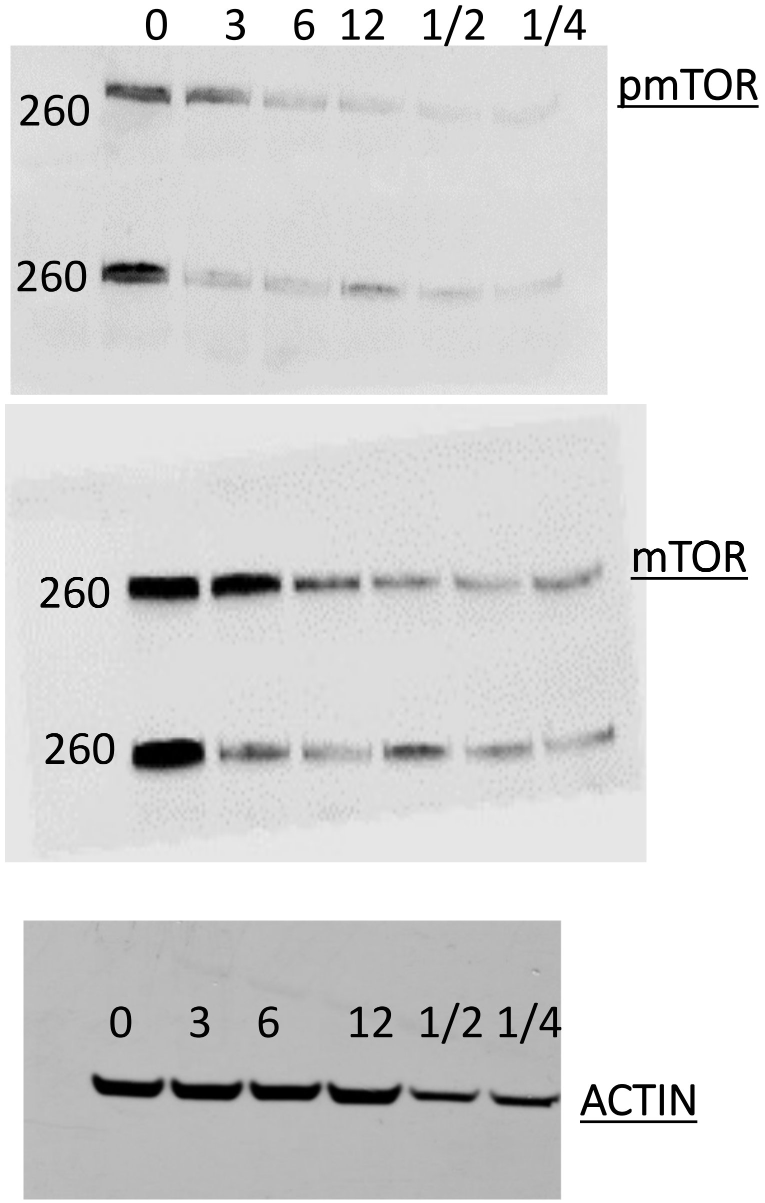

Mz-CRC 48 h

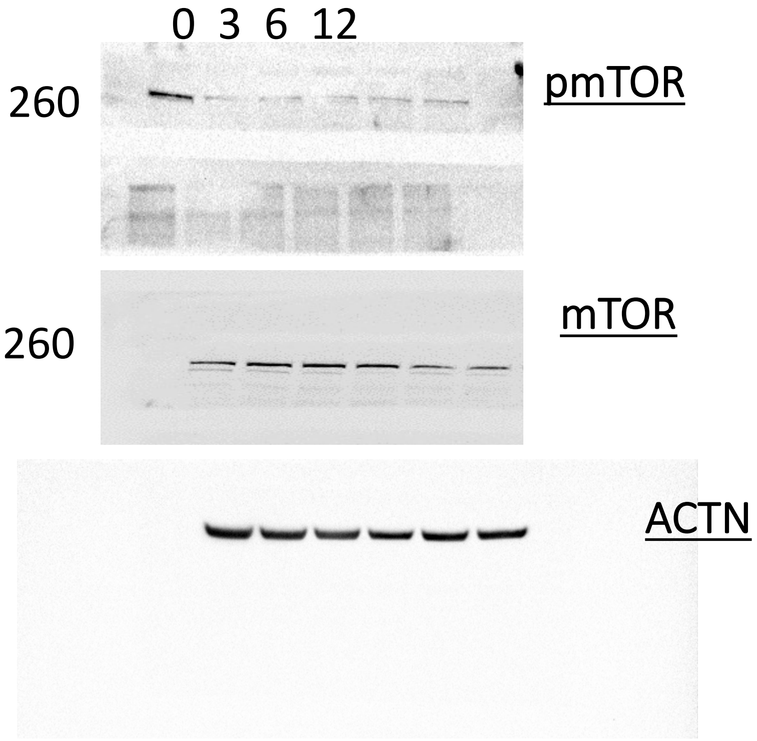

Figure 5 B

TT 48 h

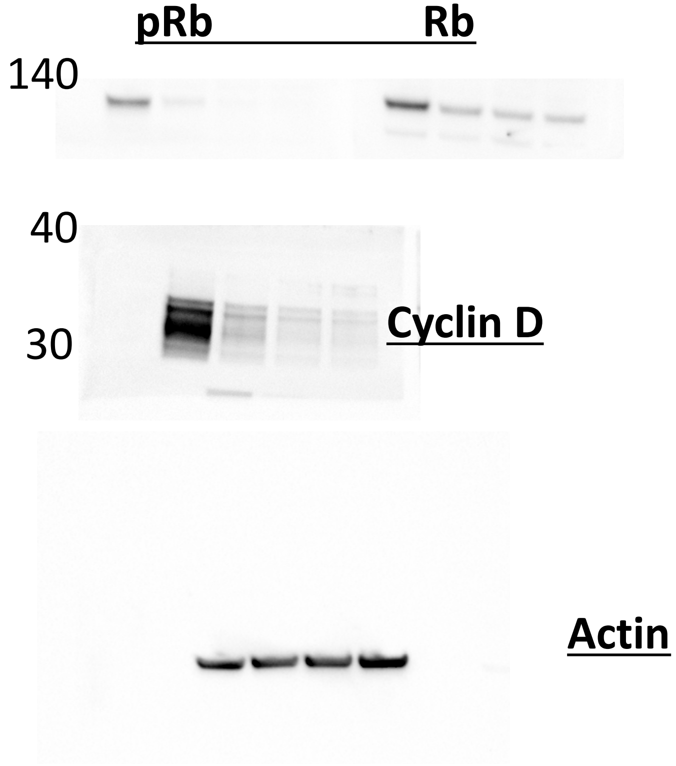

Mz-CRC -1 48 h

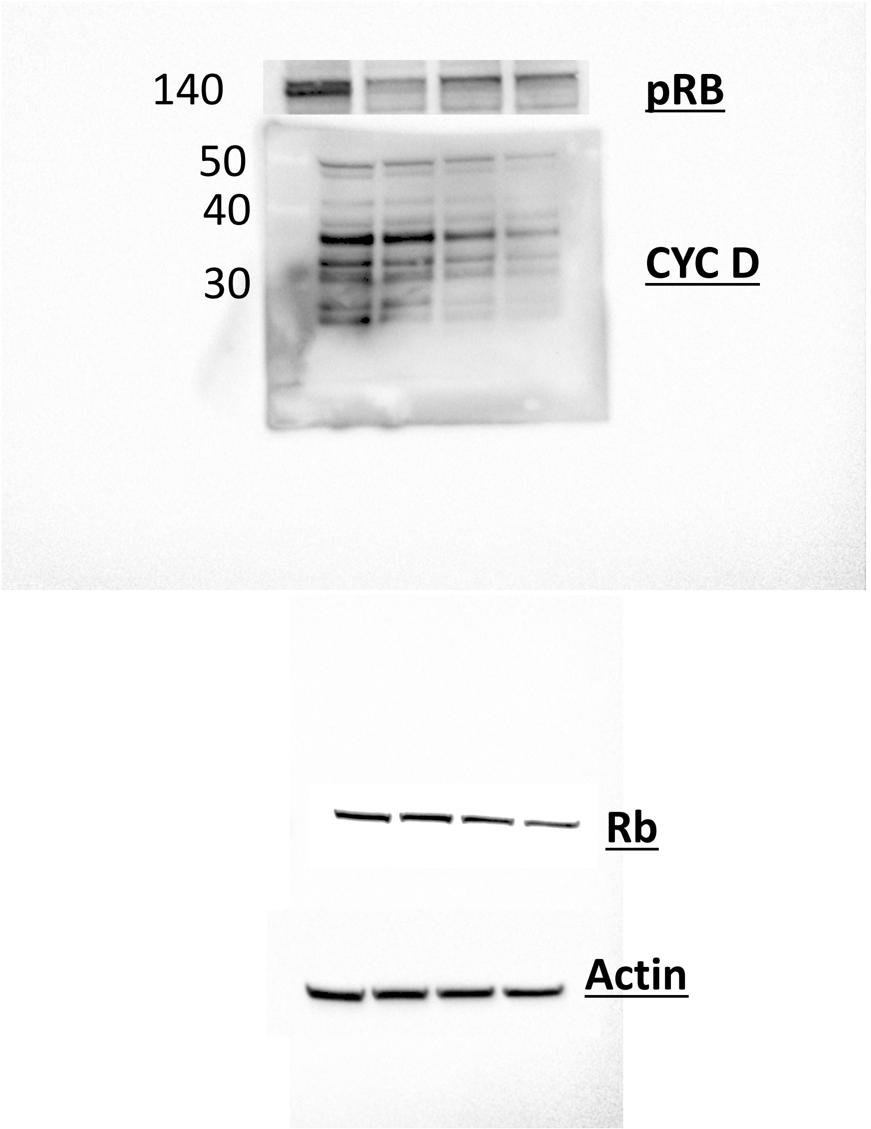

Figure 6

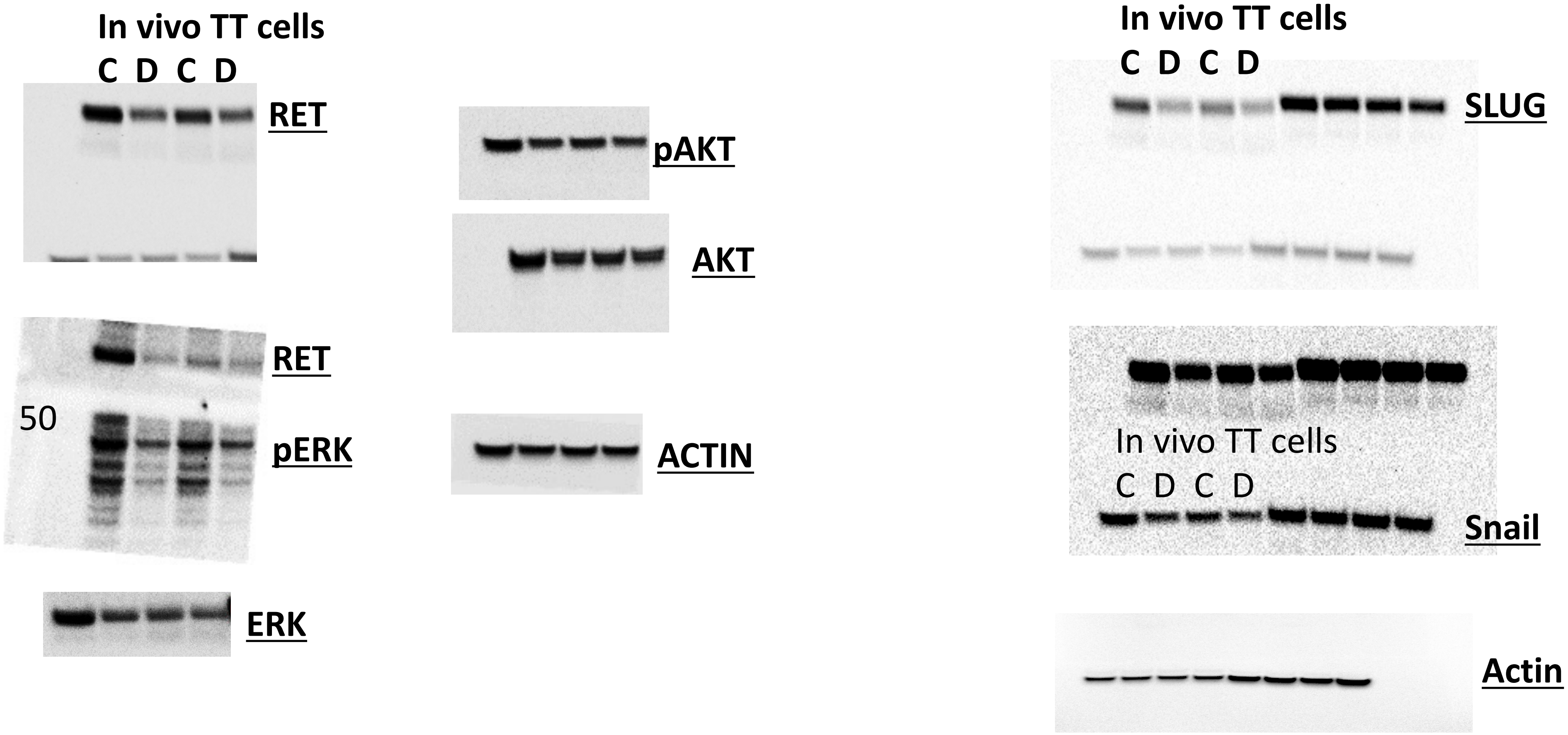

C= control
D= Datelliptium
